# Adaptogenic and Anxiolytic Effects of Ashwagandha Root Extract in Healthy Adults: A Double-blind, Randomized, Placebo-controlled Clinical Study

**DOI:** 10.7759/cureus.6466

**Published:** 2019-12-25

**Authors:** Jaysing Salve, Sucheta Pate, Khokan Debnath, Deepak Langade

**Affiliations:** 1 Internal Medicine, Risk Care Hospital/Prakruti Care Hospital/Jupiter Hospital, Thane, IND; 2 Clinical Research, Clinsearch Healthcare Solutions, Thane, IND; 3 Family Medicine, Prakruti Hospital, Mumbai, IND; 4 Pharmacology, D.Y. Patil University School of Medicine, Navi Mumbai, IND

**Keywords:** adaptogen, stress, anxiety, ashwagandha, withania somnifera, perceived stress scale, cortisol

## Abstract

Background

Stress, anxiety and impeded sleep are a frequent feature of life in modern societies. Across socio-economic strata, stress, anxiety and ineffective sleep detract from healthful living and serve as precursors of various ailments. The use of herbs to offset these antecedents and outcomes has greatly increased in recent years. Ashwagandha, an adaptogenic Ayurvedic herb, has been often used to combat and reduce stress and thereby enhance general wellbeing. While there have been other studies documenting the use of Ashwagandha for stress resistance, this is the first study to use a high-concentration root extract while also varying the dosage substantially. Therefore, this is the first study to offer insight into dose-response of a high concentration root extract.

Material and methods

In this eight-week, prospective, randomized, double-blind, placebo-controlled study, the stress-relieving effect of Ashwagandha root extract was investigated in stressed healthy adults. Sixty male and female participants with a baseline perceived stress scale (PSS) score >20 were randomized to receive capsules of Ashwagandha extract 125 mg, Ashwagandha extract 300 mg or identical placebo twice daily for eight weeks in a 1:1:1 ratio. Stress was assessed using PSS at baseline, four weeks and eight weeks. Anxiety was assessed using the Hamilton-Anxiety (HAM-A) scale and serum cortisol was measured at baseline and at eight weeks. Sleep quality was assessed using a seven-point sleep scale. A repeat measures ANOVA (general linear model) was used for assessment of treatment effect at different time periods. Post-hoc Dunnett’s test was used for comparison of two treatments with placebo.

Results

Two participants (one each in 250 mg/day Ashwagandha and placebo) were lost to follow-up and 58 participants completed the study. A significant reduction in PSS scores was observed with Ashwagandha 250 mg/day (P < 0.05) and 600 mg/day (P < 0.001). Serum cortisol levels reduced with both Ashwagandha 250 mg/day (P < 0.05) and Ashwagandha 600 mg/day (P < 0.0001). Compared to the placebo group participants, the participants receiving Ashwagandha had significant improvement in sleep quality.

Conclusion

Ashwagandha root aqueous extract was beneficial in reducing stress and anxiety.

## Introduction

Stress is a normal and natural reaction to a potentially precarious situation. A growing number of reports on stress and anxiety compelled us to seek potential medical and alternative solutions that might aid to lead a life devoid of stress and anxiety. The phenomenon of biological and genetic development considers stress and anxiety as a defensive mechanism that aids in a “fight-or-flight” response on a relevant situation. A stress response is a well-developed and evolved physiological and neurological phenomenon that becomes vital with the interplay of environmental, chemical and psychological conditions. Such responses are mandatory for the survival of an individual during a critical condition [1]. On the contrary, persistent stress response due to the environmental and social reasons may aid in developing complicated health issues such as cardiovascular disorders, hypertension, depression, panic attacks, impaired memory and cognition, digestive problems, fatigue syndrome and autoimmune disorders [2-3].

Stress can be either acute or chronic. Acute stress imparts transient physiological changes which are reversible conditions if proper treatment is done on time and homeostasis is attained within a limited period. On the other hand, chronic stress or long-time persisted stress can induce irreversible health issues with serious health damages such as metabolic syndromes, obsessive-compulsive disorder (OCD), generalized anxiety disorder (GAD), severe cardiovascular issues, hypertension, endocrinological issues, and visceral obesity [4-5].

A large group of medications is dedicated as a probable solution to persistent stress and anxiety in modern medicine. Reports suggest that such medication often turns as a source of addiction due to the altered or adaptive behavior of the subject [6-7]. Stress is associated with altered hormonal secretions of cortisol, adrenaline, and norepinephrine. Chronic stress is known to deviate the normal sleep-wake cycle by impacting the level of circadian cortisol.

Ancient Ayurvedic medicine has a remarkable solution for such lifestyle-induced biological and psychological conditions. Adaptogens are herbs that improve the responses to stress and help the body adapt by normalizing physiological processes in times of increased stress. Ideally, an adaptogen must reduce stress-induced damage, be safe, must exhibit stimulating effects, must be innocuous, must not perturb any bodily functions and must be devoid of any negative effects such as withdrawal symptoms. Adaptogens exert their stress-protective effect by regulating homeostasis via several mechanisms of action associated with the hypothalamic pituitary adrenal (HPA) axis, and also by controlling key mediators of the stress response, such as heat shock proteins (Hsp70), stress-activated c-Jun N-terminal protein kinase (JNK-1), cortisol, and nitric oxide (NO) [8-9].

In traditional Ayurveda and Unani systems of medicine, the roots of Ashwagandha have a long history of use as an adaptogen. Ashwagandha (*Withania somnifera* (L.) Dunal) is a member of the Solanaceae family of plants. Maintaining general wellbeing and improvement of vitality has been the primary importance of this “Rasayana”. Such adaptogens are efficacious in eradicating fatigue and its molecular mechanism has been explored as well [10]. Pharmacological studies have confirmed that Ashwagandha is a multipurpose herb and has anti-inflammatory, neuroprotective, adaptogenic, memory-enhancing, hematopoietic, sleep-inducing and anxiolytic properties [11-13,8,14]. A study conducted on mice using the aqueous suspensions of the powdered Ashwagandha root exhibited anti-stress activity [15]. Senthil et al. reported the free radical scavenging properties of Ashwagandha root and the presence of various phenolic compounds and flavonoids that induce antioxidant activity [16]. Since ancient times, this herb is claimed to be safe when used within the recommended dosages and formulations. Several clinical studies performed using Ashwagandha for various clinical conditions validated that it was well-tolerated and safe.

In overweight and obese adults with chronic stress, Ashwagandha root extract has been reported to improve the mental well-being, eating behaviors and reduce stress through its adaptogenic properties along with maintaining the normal endocrinological balance [17]. In addition, it was also reported to maintain an adequate range of testosterone, enhancing cognitive abilities in people with mild cognitive impairment, and boosting cardiorespiratory endurance [18-20].

The present study aimed to evaluate the effect of an aqueous Ashwagandha root extract in reducing stress and anxiety in adults. While there have been other studies documenting the use of Ashwagandha for stress resistance and sleep quality, this is the first study to use a high-concentration root extract comparing its efficacy in a low dose with the normally recommended dose. Therefore, this is the first study to offer insight into dose-response of a high concentration root extract for impact on sleep quality, psychometric stress scales and serum cortisol as a biomarker of stress. For this reason, this study may be of interest to researchers in alternative medicine seeking herbal remedies for sleep improvement and stress reduction.

## Materials and methods

Study design

This trial was a prospective, double-blind, randomized, placebo-controlled study conducted at Risk Care Hospital, Thane, Maharashtra, India. The study comprised of three arms, with two arms of the treatment group having two dosages of Ashwagandha root extract and one placebo group. A dose of 250 mg/day and 600 mg/day Ashwagandha root extract was used for the treatment groups respectively and the placebo group was given 250 mg/day starch for the entire study duration of eight weeks.

Ethical considerations

The study protocol was approved by the Institutional Ethics Committee (IEC Ref. No. DYP/IEC/04-007-A/2018, dated: 04/11/2018). The study was conducted in accordance with the Declaration of Helsinki (2013 amendment) and Good Clinical Practice (GCP) guidelines and Ethical Guidelines for Biomedical Research on Human Subjects, issued by the Indian Council of Medical Research (ICMR), were followed [21].

Study participants

Participants of either gender between 18-55 years of age were screened for study eligibility based on the inclusion and exclusion criteria. Participants were explained in detail about the objective of the study and written informed consent was obtained from all the participants before the commencement of the study.

Inclusion and exclusion criteria

Participants with a Perceived Stress Scale (PSS) score >=20 and without any other psychiatric conditions were enrolled and randomized. Participants having any chronic physical, hematological, hormonal or psychiatric illness and history of substance dependence were excluded from the study. Pregnant and lactating women and those with known hypersensitivity to Ashwagandha were also not enrolled. Further, those who were taking any other herbal preparations containing Ginseng, Brahmi, Gingko Biloba or similar herbs were not considered for participation in this study.

Randomization and sample size

Participants were randomized into three (two active treatment and one placebo treatment) groups through 1 : 1 : 1 randomization and they were allocated with the numbered treatment bottles. All the bottles were randomly allocated a number using computer software and labeled identically except for the allocation number. The randomization code was maintained independently to keep the investigators blind to the treatment allocation.

Interventions

The study comprised of an initial screening visit followed by an eight-week treatment period. On Day 0, at the screening visit, the medical history of each of the participants was obtained after obtaining written consent from the subjects. The vital parameters were recorded for every participant, and symptoms of stress were assessed using the PSS. After assessing the screening parameters, each participant was randomly assigned into either of the three study groups; two study treatment groups: Ashwagandha 250, Ashwagandha 600 and one placebo group.

Participants were provided bottles containing either the active treatment component or the placebo. Each participant was instructed to consume one capsule, two times a day, after food with milk or water for the entire duration of the study. Of the two active treatment groups, one was given capsules containing 125 mg Ashwagandha root extract, and the other was given capsules containing 300 mg Ashwagandha root extract. The placebo group participants were given capsules containing 125 mg of starch. The capsules containing Ashwagandha and placebo were of exact shape, size and color, and appearance.

Investigational product

KSM-66 Ashwagandha root extract was used in this study. The product was manufactured and gifted by Ixoreal Biomed Inc., Los Angeles, California, USA (Batch #: KSM/VG/18/1085).

Assessments

Perceived Stress Scale

The Perceived Stress Scale is a classic and commonly used stress assessment instrument in clinical psychology. It contains a self-reporting questionnaire consisting of 10 items, that are ideal to assess the level of stress perceived by a participant. Further, this 10-item version of the scale (PSS-10) comprises four positive and six negative items. The negative items are intended to assess the lack of control and negative affective reactions, whereas the positive items measure the ability to cope with existing stressors. The score for each of the item is rated on a 5-point Likert scale, that is presented from 0 (*never*) to 4 (*very often*). The absolute values of the scale range from 0 to 40 with higher scores indicating higher levels of perceived stress [22].

Serum Cortisol

Serum cortisol is the most widely used biomarker to assess physiological stress. Hence, it was used to assess the effect of the Ashwagandha root extract on the cortisol level. The serum cortisol level of each participant was measured from the blood sample collected in the morning through venipuncture. The serum cortisol level was measured at the baseline, at week 4, and at week 8. The serum cortisol levels were measured in ug/dL for the present study.

Hamilton Anxiety Rating Scale (HAM-A) Assessment

The Hamilton Anxiety Rating Scale (HAM-A) was used to assess the intensity of anxiety present in each participant [23]. HAM-A consists of 14 items, each defined by a series of symptoms and measures both somatic and psychic anxiety. The scoring is performed using a scale of 0 to 4 (*Not present* to *Severe*) for each item. The absolute values for each item range from 0 to 56.

Sleep Quality

Sleep quality was assessed for each study participant using standard sleep quality questionnaire during the visits. All the participants were requested to provide feedback on their sleep quality. After waking up in the morning, the overall sleep quality as perceived by the participant was estimated through a seven-point scale. The scoring was considered as follows: 1 = *Excellent*, 2 = *Very Good*, 3 = *Good*, 4 = *Fair*, 5 = *Poor*, 6 = *Very poor* and 7 = *Extremely poor*.

Safety Assessments

Clinical safety and tolerability of the interventions were measured by analyzing the significant changes in the vital parameters and the biochemical parameters assayed. Assessment of the adverse events reports was also considered as part of the safety evaluation.

Statistical Analysis

All the participants enrolled in this study were analyzed according to their randomized group in the per-protocol dataset, regardless of compliance with the treatment or any other deviation from protocol. Efficacy was evaluated based on physiological and psychometric measures.

The obtained analysis outcome of ranking data and scores are represented here as mean with standard deviation (SD). Throughout the analysis, 95% confidence intervals (CI) were considered wherever necessary. In the present study, baseline scores were compared to the post-treatment scores for the different scales using the Friedman test within the group. The three groups were compared using the one-way Analysis of Variance (ANOVA). A comparison between the study treatment group and placebo was done through Post-hoc analysis using two-sided Dunnett T-tests.

## Results

Seventy-five participants were screened for participation in the study and 60 participants met the inclusion criteria and were enrolled to participate. Two participants dropped out of the study (one from Ashwagandha 250 and the other from the placebo group), as they did not report during follow-up (lost to follow-up). The analysis was continued using the data for the remaining 58 participants.

**Figure 1:**
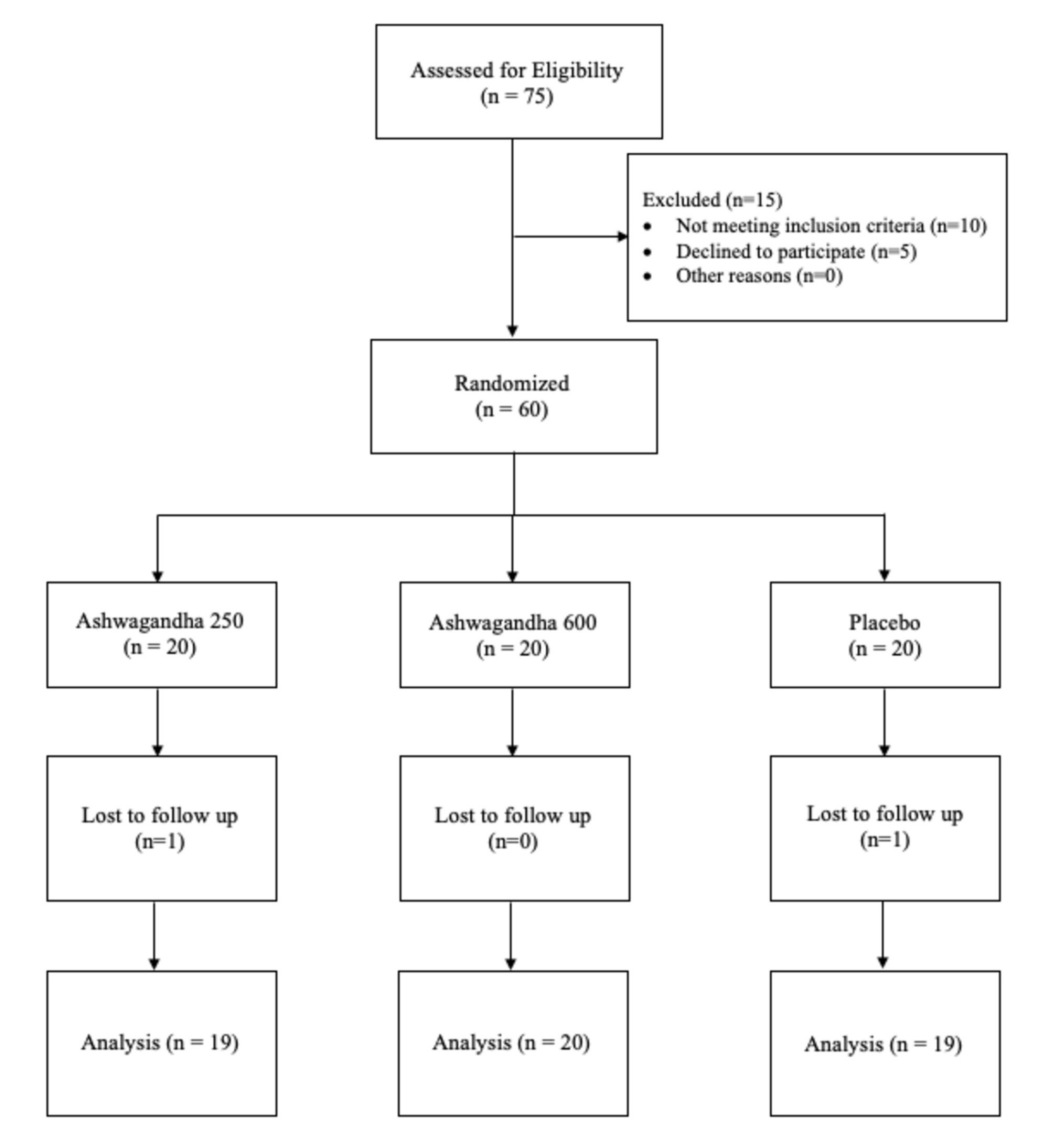
CONSORT Flow chart of participant allocation

The mean (SD) age of the participants in Ashwagandha 250, Ashwagandha 600 and placebo group were 29.65 (6.36), 32.70 (8.79), and 30.35 (6.50), respectively. The demographic characteristics of the three groups indicated that the study population was homogenous with no statistically significant differences between the groups (Figure 1).

Perceived stress scale (PSS)

Perceived stress scale was measured at the baseline, week 4, and at the end of the study. Table 1 shows that perceived stress scores declined in all three groups over the eight weeks of study, with the decrease being significantly greater among the subjects in Ashwagandha groups (Ashwagandha 250 and Ashwagandha 600) when compared to the placebo group. The baseline scores in Ashwagandha 250, Ashwagandha 600 and placebo group were 22.65 (1.75), 22.95 (1.57), and 22.70 (2.17), respectively. After eight weeks of treatment, the recorded scores were 15.00 (2.21) for Ashwagandha 250 group, 14.15 (2.62) for Ashwagandha 600 group, and 16.63 (3.13) for the placebo group.

Relative to the value at the baseline, the mean PSS was significantly lower (p < 0.05) in the Ashwagandha 250 group and also (p < 0.05) in the Ashwagandha 600 group. The reduction in mean PSS relative to the baseline was significantly higher than in the placebo group both for the Ashwagandha 250 group (p < 0.05) and in the Ashwagandha 600 group (p < 0.001). This shows that while the Ashwagandha 250 mg/day treatment is effective in reducing PSS, it is not as effective as the Ashwagandha 600 mg/day (Table 1).

**Table 1:** Perceived Stress Scale (PSS) score * and ** indicate p < 0.05 and p < 0.001 respectively for Dunnett 2-sided t-test for improvement from baseline being higher than for placebo.

	Ashwagandha 250 mg/day			Ashwagandha 600 mg/day			Placebo		
	N	Mean (SD)	95% CI	N	Mean (SD)	95% CI	N	Mean (SD)	95% CI
Baseline	20	22.65 (1.75)	21.83-23.47	20	22.95 (1.57)	22.21-23.69	20	22.70 (2.17)	21.68-23.72
Week 4	19	18.79 (2.20)	17.73-19.85	20	18.85 (2.05)	17.89-19.81	19	19.89 (2.30)	18.78-21.01
Week 8	19	15.00 (2.21) *	13.93-16.07	20	14.15 (2.62) **	12.92-15.38	19	16.63 (3.13)	15.12-18.14

Serum cortisol (mcg/dL)

At the end of the study, there was a significant decrease in serum cortisol levels in the Ashwagandha treatment groups compared to the placebo group. Table 2 summarizes the results obtained for serum cortisol assessment. The three groups (two Ashwagandha and one placebo) were having similar baseline scores.

Relative to the value at baseline, the mean cortisol level was statistically significantly lower (p < 0.05) in the Ashwagandha 250 group and also (p < 0.05) in the Ashwagandha 600 group. The decrease in the mean cortisol, relative to the baseline was statistically significantly higher both in the Ashwagandha 250 group (p < 0.05) and in the Ashwagandha 600 group (p < 0.0001), than in the placebo group. This indicates that while Ashwagandha 250 mg/day treatment is effective in reducing the cortisol level, it is not as effective as Ashwagandha 600 mg/day (Table 2).

**Table 2:** Serum cortisol (mcg/dL) * and *** indicate p < 0.05 and p < 0.0001 respectively for Dunnett 2-sided t-test for improvement from baseline being higher than for placebo.

	Ashwagandha 250			Ashwagandha 600			Placebo		
	N	Mean (SD)	95% CI	N	Mean (SD)	95% CI	N	Mean (SD)	95% CI
Baseline	20	16.30 (4.72)	14.09-18.51	20	16.12 (3.97)	14.26-17.98	20	16.15 (4.80)	13.90-18.39
Week 4	19	14.74 (4.83)	12.41-17.07	20	14.30 (3.75)	12.55-16.06	19	15.52 (4.57)	13.31-17.73
Week 8	19	13.61 (4.57) *	11.41-15.82	20	10.86 (3.80) ***	9.08-12.64	19	15.52 (4.57)	13.32-17.72

Hamilton Anxiety Rating Scale (HAM-A) analysis

The change in HAM-A scores in the three groups was shown in Table 3. At the baseline, the recorded HAM-A scores were 23.05 (3.22), 24.10 (3.21), and 23.32 (3.09) for Ashwagandha 250, Ashwagandha 600, and placebo groups, respectively. At the end of the study, the scores were recorded as 20.05 (2.85), 20.15 (3.66), and 21.42 (3.27), respectively.

Relative to the value at baseline, the mean HAM-A was not significantly lower in the Ashwagandha 250 group, but statistically significantly lower (p < 0.05) in the Ashwagandha 600 group. The reduction in the mean HAM-A relative to the baseline was statistically significantly higher in the Ashwagandha 600 group (p < 0.0001) than in the placebo group. The reduction in the HAM-A was not statistically significant in the Ashwagandha 250 group when compared to the placebo group. The results show that Ashwagandha 600 mg/day treatment is effective in reducing anxiety (Table 3).

**Table 3:** Hamilton Anxiety Rating Scale (HAM-A) *** indicates p < 0.0001 for Dunnett 2-sided t-test for improvement from baseline being higher than for placebo.

	Ashwagandha 250			Ashwagandha 600			Placebo		
	N	Mean (SD)	95% CI	N	Mean (SD)	95% CI	N	Mean (SD)	95% CI
Baseline	20	23.05 (3.22)	21.50-24.61	20	24.10 (3.21)	22.60-25.60	20	23.32 (3.09)	21.83-24.81
Week 4	19	21.37 (3.05)	19.89-22.84	20	22.10 (3.56)	20.43-23.77	19	22.26 (3.26)	20.69-23.84
Week 8	19	20.05 (2.85)	18.68-21.43	20	20.15 (3.66) ***	18.44-21.86	19	21.42 (3.27)	19.84-23.00

Sleep quality assessment

**Figure 2:**
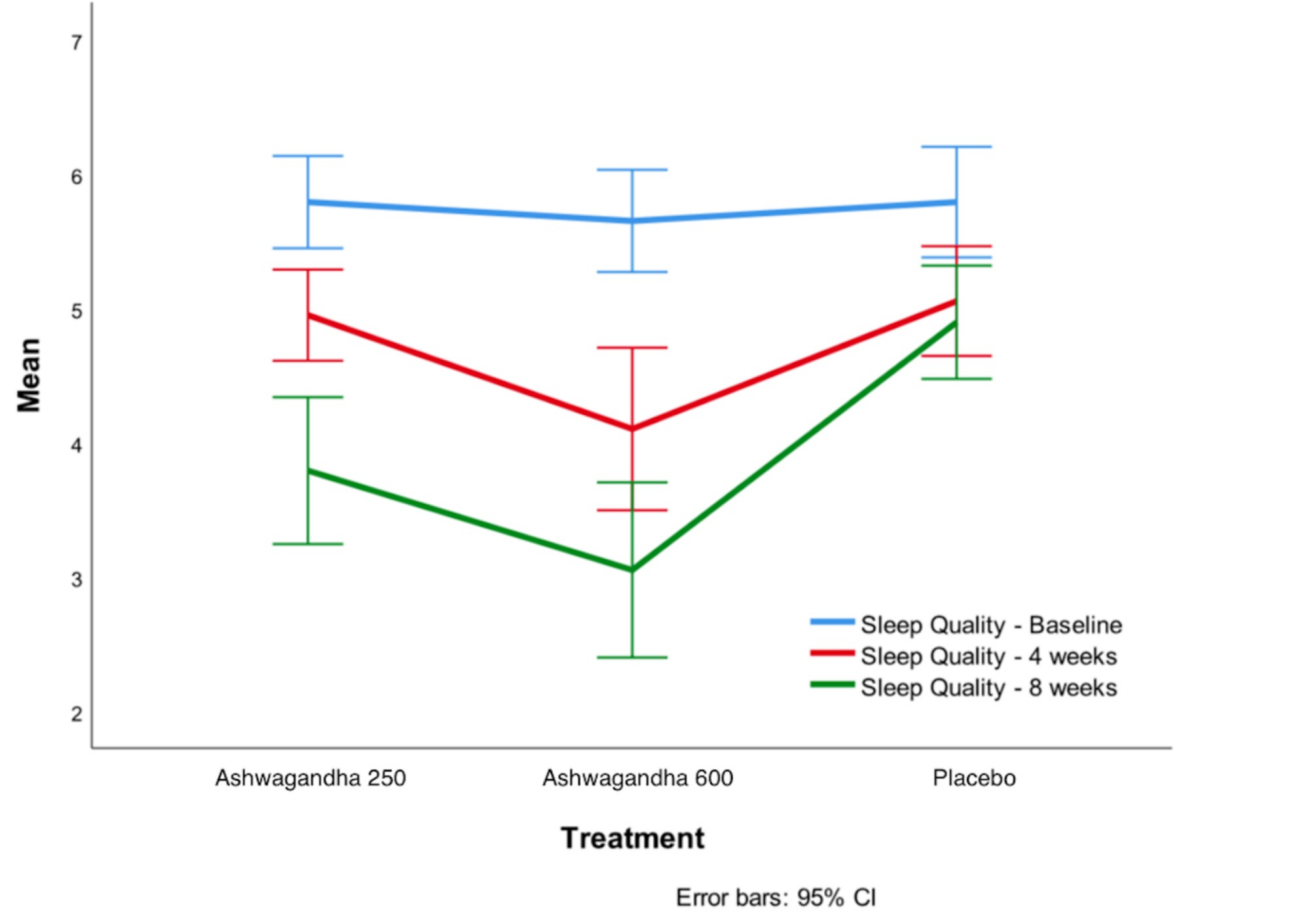
Sleep quality scores at baseline, week 4 and week 8

Over the eight weeks, there was a significant improvement in sleep quality, with both Ashwagandha 250 mg and Ashwagandha 600 mg compared to the placebo group (Figure 2). Table 4 represents the results obtained over the eight weeks for quality of sleep assessment using ANOVA followed by post hoc analysis. Relative to the value at baseline, the mean sleep quality score was statistically significantly higher (p < 0.05) in the Ashwagandha 250 group and also (p < 0.05) in the Ashwagandha 600 group. The increase in the mean sleep quality score, relative to the baseline was statistically significantly higher both in the Ashwagandha 250 group (p < 0.05) and in the Ashwagandha 600 group (p < 0.0001), than in the placebo group. This shows that while Ashwagandha 250 mg/day treatment is effective in improving the quality of sleep, it is not as effective as Ashwagandha 600 mg/day.

**Table 4:** Data for quality of sleep * and ** indicate p < 0.05 and p < 0.001 respectively for Dunnett 2-sided t-test for improvement from baseline being higher than for placebo.

	Ashwagandha 250 mg/day			Ashwagandha 600 mg/day			Placebo		
	N	Mean (SD)	95% CI	N	Mean (SD)	95% CI	N	Mean (SD)	95% CI
Baseline	20	5.85 (0.74)	5.50–6.20	20	5.65 (0.81)	5.27–6.03	20	5.85 (0.87)	5.44–6.26
Week 4	19	4.95 (0.70)	4.61–5.29	20	4.10 (1.29)	3.49–4.71	19	5.05 (0.84)	4.64–5.46
Week 8	19	3.79 (1.13) *	3.24–4.34	20	3.05 (1.39) **	2.40–3.70	19	4.89 (0.87)	4.47–5.32

Adverse events

Data for adverse events were available only for 58 participants as two subjects were lost in follow-up. It was noted that Ashwagandha was well tolerated with no adverse events reported by the participants.

## Discussion

Stress and anxiety have been identified as promoters of multiple disease conditions including neurological conditions such as Alzheimer’s disease, cardiovascular problems such as hypertension and heart disease, lifestyle diseases such as diabetes and obesity and so on. More importantly, our present stressful lifestyle requires proper anxiety and behavioral management. Modern drugs have shown adverse effects and drug dependence in many earlier reports.

Ashwagandha, an Ayurvedic adaptogen, is known to have a remarkable impact on the stress that may provide the well-cherished outcome in this aspect and help in restoring a normal lifestyle with reduced stress and prevent the onset of several life-threatening disease conditions.

The present randomized, double-blind, placebo-controlled clinical study evaluated the effect of an aqueous root extract of Ashwagandha in 58 participants having stress and anxiety. The results indicated that the treatment with Ashwagandha root extract was considerably effective compared to the placebo.

Several studies have been carried out in the last few years on the adaptogenic effects of Ashwagandha. The anti-stress effect of alcoholic root extract of *Withania somnifera *was assessed in mice through evaluating the swimming performance in the water. The results showed that *Withania somnifera *induced a stage of nonspecific increased resistance during stress. Chandrasekhar et al. studied the safety and efficacy of high concentration and full-spectrum Ashwagandha root extract in reducing stress and anxiety in 64 subjects in a 60-day long clinical trial [24]. The study also reported that a significant reduction was observed in all the stress assessment scale scores and the levels of serum cortisol when compared to the placebo group. Another report suggests that pre-treated rats with an aqueous suspension of Ashwagandha root extract were successful in decreasing the adrenal cortisol and ascorbic acid levels in animals subjected to swimming stress [25]. Another study evaluated the effect of ethanolic extract of *Withania somnifera *roots against acute stress induced in mice showing that pre-treatment of animals with *Withania somnifera* extract improved the swimming duration in mice. Further, the treatment with Ashwagandha significantly restored the stress-induced alterations in plasma cortisol, blood glucose, and triglyceride levels [26]. Similar effects of Ashwagandha root extract were also seen in stressed and overweight adults [17].

Sleep quality always has an impact on our overall health conditions. Lack of sleep can induce multiple disease conditions in an individual, which is well established by several scientific reports. Stress, anxiety, and sleep have an unavoidable relation. Better sleep aids in reducing stress and anxiety whereas poor sleep quality may induce anxiety and stress. Therefore, assessment of sleep quality is mandatory in relation to stress management.

In this current clinical study, the effect of a lower dose of Ashwagandha root extract (Ashwagandha 250 mg/day) was evaluated and compared with the standard dosage of Ashwagandha (Ashwagandha 600 mg/day) and placebo in stressed individuals. The subject group receiving Ashwagandha 250 mg showed a statistically significant reduction in the stress levels, assessed using the Perceived Stress Scale (PSS) and serum cortisol. Significant improvement was also noticed for the sleep quality of the participants which is an important indicator of better stress management. The reduction in anxiety was not statistically significant when compared to the placebo. The study group that received Ashwagandha 600 mg showed a statistically significant reduction in all the components used for stress and anxiety and statistically significant improvement in sleep quality.

The present study provides valuable insight with regard to the use of Ashwagandha root extract as a stress managing adaptogen apart from its other specific health-related application. Comparative dosage evaluation also provided some important information regarding the ability of the extract.

Limitations

A study with a larger population involving a wider cross-section of participants with regard to age groups, occupation, and socio-economic background would provide more conclusive results. Moreover, the study duration should also be increased in future research to evaluate the long-term effects of Ashwagandha root extract in varying doses. Additional parameters like serotonin, DHEA-s and other related hormonal levels that are altered during stress need to be assessed also.

## Conclusions

Ashwagandha is a medically important herb and has a proven impact on human health. The findings from this study suggest that eight weeks supplementation of aqueous Ashwagandha root extract was associated with a significant reduction of stress levels in individuals and improved the overall quality of life. Hence, the use of this herb as a supplement for stress and anxiety management could be an excellent alternative option. Further studies conducted with a larger cohort and in diverse populations and with more biochemical, physiological and psychological evaluation may confirm the present findings.
